# Deep learning for differential diagnosis of malignant hepatic tumors based on multi-phase contrast-enhanced CT and clinical data

**DOI:** 10.1186/s13045-021-01167-2

**Published:** 2021-09-26

**Authors:** Ruitian Gao, Shuai Zhao, Kedeerya Aishanjiang, Hao Cai, Ting Wei, Yichi Zhang, Zhikun Liu, Jie Zhou, Bing Han, Jian Wang, Han Ding, Yingbin Liu, Xiao Xu, Zhangsheng Yu, Jinyang Gu

**Affiliations:** 1 Department of Bioinformatics and Biostatistics, School of Life Sciences and Biotechnology, Shanghai Jiao Tong University, Shanghai, 200240 China; 2 Department of Transplantation, Xinhua Hospital Affiliated To Shanghai Jiao Tong University School of Medicine, Shanghai, 200092 China; 3 Department of Hepatobiliary and Pancreatic Surgery, The Center for Integrated Oncology and Precision Medicine, Affiliated Hangzhou First People’s Hospital, Zhejiang University School of Medicine, Hangzhou, 310006 China; 4 Department of Biliary-Pancreatic Surgery, Renji Hospital Affiliated To Shanghai Jiao Tong University School of Medicine, Shanghai, 200120 China; 5 SJTU-Yale Joint Center for Biostatistics and Data Science, Shanghai Jiao Tong University, Shanghai, 200240 China; 6 Clinical Research Institute, Shanghai Jiao Tong University School of Medicine, Shanghai, 200025 China

**Keywords:** Artificial intelligence, Liver cancer, Contrast-enhanced CT, Computer-assisted diagnosis, Multimodal data

## Abstract

**Background:**

Liver cancer remains the leading cause of cancer death globally, and the treatment strategies are distinct for each type of malignant hepatic tumors. However, the differential diagnosis before surgery is challenging and subjective. This study aims to build an automatic diagnostic model for differentiating malignant hepatic tumors based on patients’ multimodal medical data including multi-phase contrast-enhanced computed tomography and clinical features.

**Methods:**

Our study consisted of 723 patients from two centers, who were pathologically diagnosed with HCC, ICC or metastatic liver cancer. The training set and the test set consisted of 499 and 113 patients from center 1, respectively. The external test set consisted of 111 patients from center 2. We proposed a deep learning model with the modular design of *SpatialExtractor-TemporalEncoder-Integration-Classifier* (STIC), which take the advantage of deep CNN and gated RNN to effectively extract and integrate the diagnosis-related radiological and clinical features of patients. The code is publicly available at https://github.com/ruitian-olivia/STIC-model.

**Results:**

The STIC model achieved an accuracy of 86.2% and AUC of 0.893 for classifying HCC and ICC on the test set. When extended to differential diagnosis of malignant hepatic tumors, the STIC model achieved an accuracy of 72.6% on the test set, comparable with the diagnostic level of doctors’ consensus (70.8%). With the assistance of the STIC model, doctors achieved better performance than doctors’ consensus diagnosis, with an increase of 8.3% in accuracy and 26.9% in sensitivity for ICC diagnosis on average. On the external test set from center 2, the STIC model achieved an accuracy of 82.9%, which verify the model’s generalization ability.

**Conclusions:**

We incorporated deep CNN and gated RNN in the STIC model design for differentiating malignant hepatic tumors based on multi-phase CECT and clinical features. Our model can assist doctors to achieve better diagnostic performance, which is expected to serve as an AI assistance system and promote the precise treatment of liver cancer.

**Supplementary Information:**

The online version contains supplementary material available at 10.1186/s13045-021-01167-2.

## To the editor

**Fig. 1:**
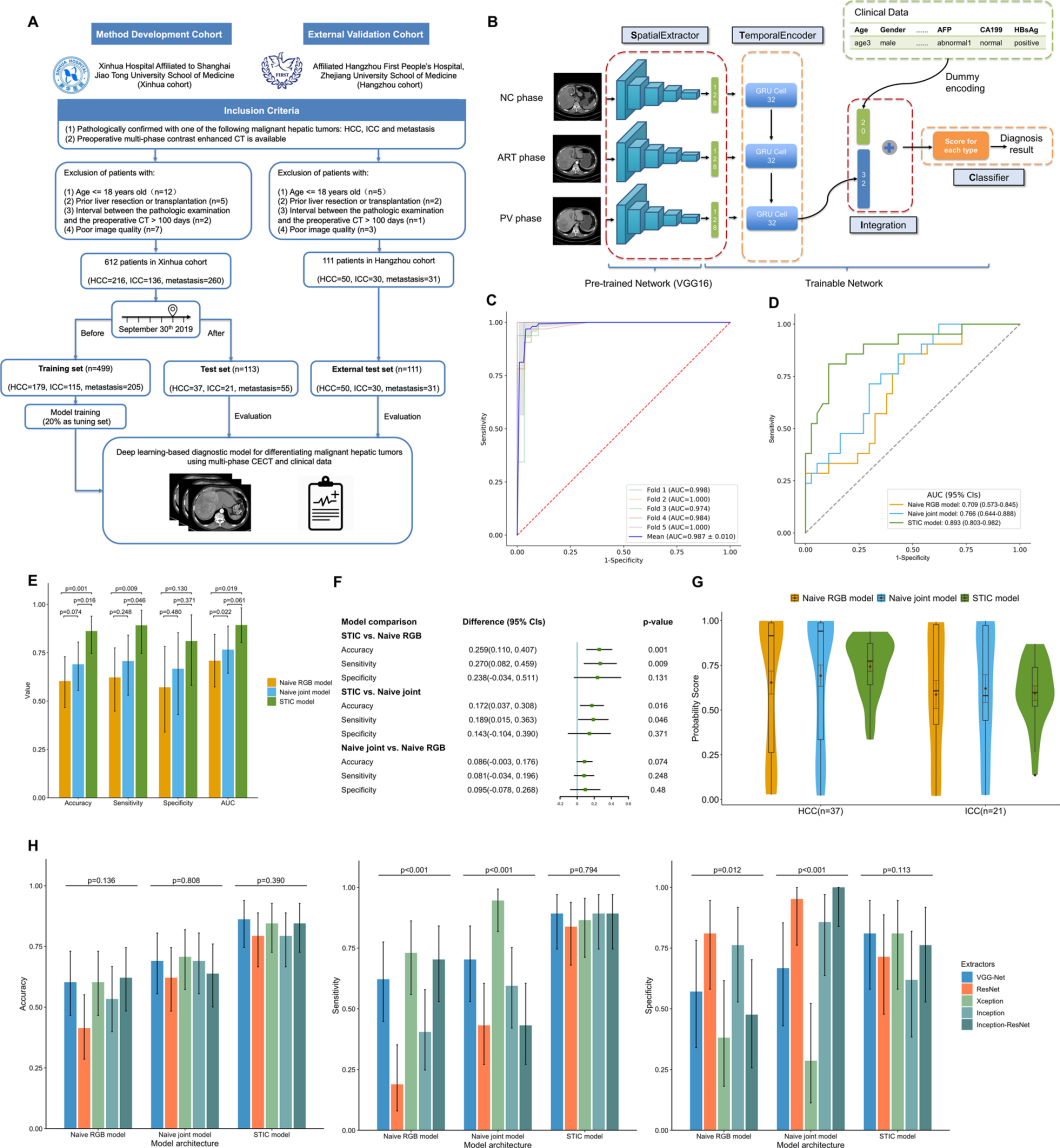
The flowchart of dataset setup, the architecture of the STIC model and the performance on primary malignant hepatic tumors classification. **A** This study consisted of 612 patients in method development cohort and 111 patients in external validation cohort, who were pathologically diagnosed with HCC, ICC or metastatic liver cancer. **B** The STIC model contains four different modules. *SpatialExtractor* module is a deep CNN that uses convolutional layers to extract detailed spatial features of CECT images. *TemporalEncoder* module uses gated RNN to mine the changing pattern among different CECT phases. In the *Integration* module, the *TemporalEncoder* module is concatenated with the vector of encoded dummy clinical variables. Finally, in the *Classifier* module, the *Integration* output is passed through the softmax activation function to implement the classification task. **C** The ROC curves of five-fold cross-validation of the STIC model for classifying benign and malignant hepatic tumors in the preliminary study, where the mean ROC curve was obtained by interpolation of the ROC curves of each fold, with mean AUC of 0.987. **D** Comparison of the performance for differencing HCC and ICC on the test set by ROC curve analysis. The AUC of the STIC model was 0.893 (95% CIs, 0.803–0.982), which was much higher than 0.709 (95% CIs, 0.573–0.845) in the Naive RBG model and 0.766 (95% CIs, 0.644–0.888) in the Naive joint model. **E** Among three models, the STIC model produced the best performance in distinguishing two primary malignant hepatic tumors, with accuracy of 86.2% (95% CIs, 74.6%-93.9%), sensitivity of 0.892 (95% CIs, 0.746–0.970) and specificity of 0.810 (95% CIs, 0.581–0.946), where sensitivity and specificity are defined by viewing HCC as positive and ICC as negative. The error bars represent 95% CIs calculated by Wald Z Method with Continuity Correction for accuracy, sensitivity and specificity and by DeLong method for AUC. **F** Using McNemar’s Chi-squared test, the STIC model outperformed the Naive RBG model with an increase of 25.9% (95% CIs 11.0%-40.7%, *p* value = 0.001) in accuracy and 0.270 (95% CIs 0.082–0.459, *p* value = 0.009) in sensitivity. It also outperformed the Naive joint model with an increase of 17.2% (95% CIs 3.7%-30.8%, *p* value = 0.016) in accuracy and 0.189 (95% CIs 0.015–0.363, *p* value = 0.046) in sensitivity. **G** The distribution of the predicted score for HCC and ICC according to three models. For two benchmark models, the score predicted had much wider distribution. Our proposed STIC model had a more concentrated distribution of predicted scores for both HCC and ICC. **H** Comparison of the performance of the STIC model and two benchmark models using different extractor’s backbone for binary classification of primary malignant hepatic tumors. Using Cochran’s Q test, there were no significant differences in the diagnostic level among STIC models with different extractor’s backbone. For Naïve RGB models with different extractor’s backbone, there were significant differences in sensitivity (*p* value < 0.001) and specificity (*p* value = 0.012). For Naïve joint models with different extractor’s backbone, there were also significant differences in sensitivity (*p* value < 0.001) and specificity (*p* value < 0.001)

Liver cancer is the sixth most commonly diagnosed cancer and the third leading cause of cancer death in the world according to 2020 global cancer statistics [1]. A substantial number of malignant liver tumors are primary tumors, including HCC and ICC [2]. In clinical settings, the metastasis of tumors to the liver is also frequently encountered [3]. The treatment regimen for the different subtypes of hepatic tumors is all distinct [4], and multi-phase CECT has become the primary tool for diagnosis of hepatic tumors before surgery [5]. However, the differential diagnosis of malignant hepatic tumors is challenging, and misdiagnosis prior to surgery can mislead the treatment decision. An automated diagnostic model is desirable to be developed, which can assist doctors in hepatic tumors diagnosis, reduce observer variations and improve diagnostic efficiency. Few preliminary studies utilized deep learning to differentiate hepatic tumors [6–9], but they lacked detailed classification for malignant hepatic tumors, especially for ICC. Herein, we proposed a novel deep learning model, which was specifically customized for the differential diagnosis of malignant hepatic tumors based on patients’ preoperative multi-phase CECT and clinical features. All 723 patients enrolled in our study were pathologically confirmed with one of the following malignant hepatic tumors: HCC, ICC and metastatic liver cancer (Fig. 1A). The training and test sets were split, with 499 and 113 patients from center 1, respectively. The external test set consisted of 111 patients from center 2, which was considered as additional verification (Additional file 2: Table S1). Our proposed model has the modular design of *SpatialExtractor-TemporalEncoder-Integration-Classifier* (STIC), which takes the preprocessed multi-phase CECT images (Additional file 2: Figure S1) and corresponding encoded clinical features (Additional file 2: Table S2) as input, and finally output the score for each category (Fig. 1B). The Python code implementing the model is available at https://github.com/ruitian-olivia/STIC-model. The materials and methods are shown in detail in the Additional file 1.

## Differentiation between benign and malignant hepatic tumors: a preliminary study

As a preliminary study, we trained the STIC model for benign and malignant hepatic tumors classification on a relatively small dataset, with 152 pathologically confirmed benign hepatic tumors and 159 malignant hepatic tumors (Additional file 2: Table S3). Using five-fold cross-validation, our proposed model achieved the mean accuracy of 93.2% and AUC of 0.987 (Fig. 1C and Additional file 2: Table S4), which demonstrated the ideal classification ability of the STIC model.

## Binary classification of primary malignant hepatic tumors

We then trained the STIC model for differentiating two primary malignant hepatic tumors on the training set and achieved the accuracy of 86.2% on the test set. For comparison, we also built two benchmark models, Naïve RGB model and Naïve joint model (Additional file 2: Figure S2), which used channel assignment strategy reported by previous studies [7]. According to ROC analysis, the STIC model achieved better performance than two benchmark models, with AUC of 0.893 (Fig. 1D). In terms of accuracy, sensitivity and specificity, the STIC models also produced the best performance (Fig. 1E and Additional file 2: Table S5), with significant increase compared with two benchmark models (Fig. 1F). The scores predicted by the STIC model had more concentrated distribution both for HCC and ICC (Fig. 1G). Using different extractor’s backbone in *SpatialExtractor* module, the STIC model’s performance always remained stable without significant changes. However, the performance of two benchmark models using different extractor’s backbone fluctuates greatly, failing to maintain a balance between sensitivity and specificity (Fig. 1H and Additional file 2: Table S6). The combination of deep CNN and gated RNN in two modules of our STIC model can effectively extract the spatial and temporal features of multi-phase CECT, which is more powerful than the channel assignment strategy used in benchmark models.

## Multinomial classification of malignant hepatic tumors and performance of the STIC-assisted diagnosis

**Fig. 2:**
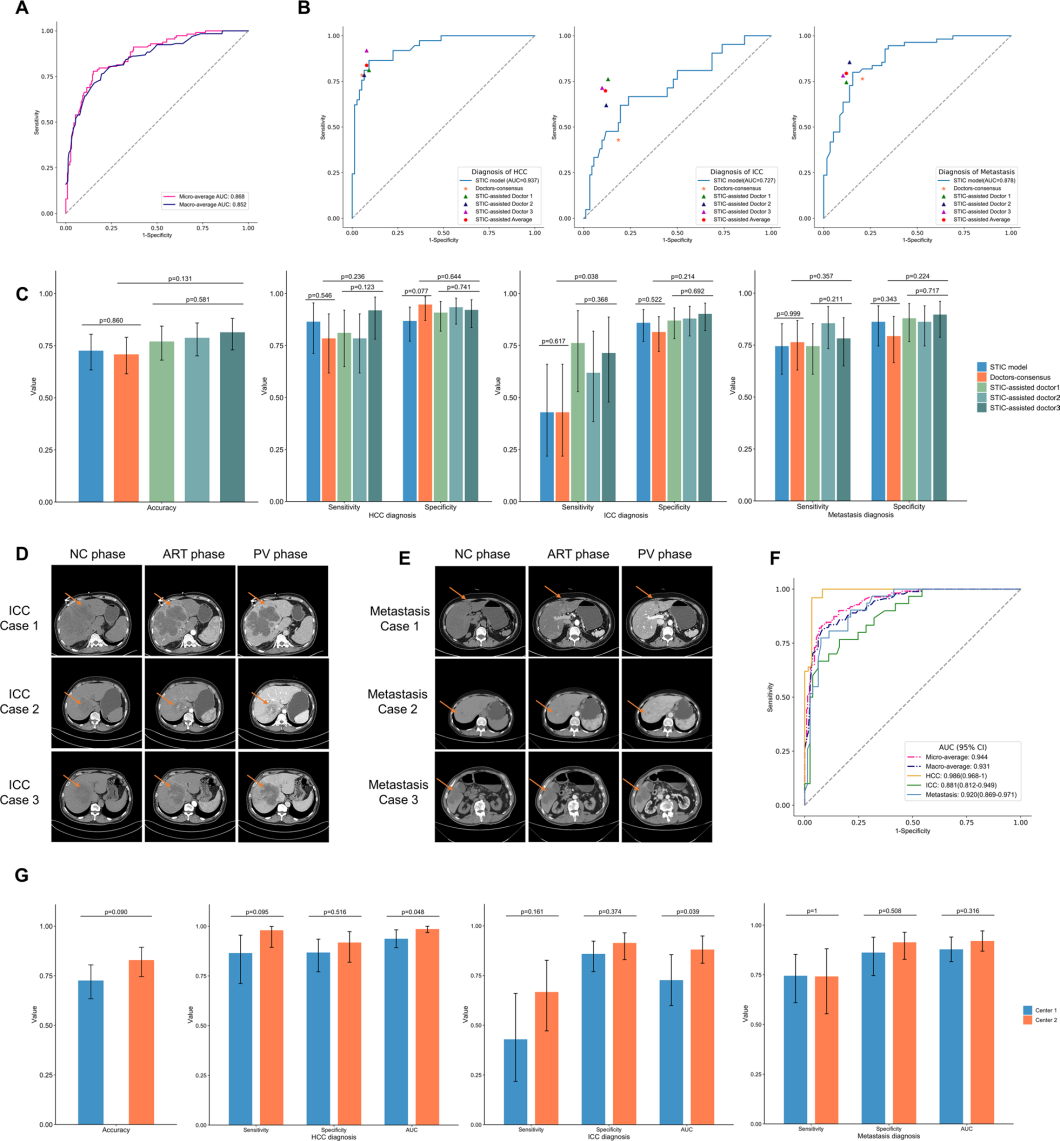
Model’s performance on the multinomial classification of malignant hepatic tumors **A** Micro-average and macro-average ROC curves of the STIC model for differentiating HCC, ICC and metastasis on the test set. **B** The ROC curves of the STIC model for HCC, ICC, metastasis diagnosis on the test set and corresponding diagnosis points of doctors’ consensus and three STIC-assisted doctors. The orange star represents the diagnostic performance of doctors’ consensus. Three triangles with different colors represent the diagnostic performance of three STIC-assisted doctors, respectively, and the red pentagon represents the average diagnostic level of these three doctors. For the ICC diagnosis, the performance of doctors’ consensus diagnosis was below the ROC curve of the STIC model, and the performances of three STIC-assisted doctors were all above the ROC curve. **C** The total accuracy of the STIC model was 72.6% (95% CIs, 63.4%-80.5%), and the total accuracy of the doctors’ consensus was 70.8% (95% CIs, 61.5%-79.0%). Three STIC-assisted doctors achieved the total accuracy of 77.0% (95% CIs, 68.1%-84.4%), 78.8% (95% CIs, 70.1%-85.9%) and 81.4% (95% CIs, 73.0%-88.1%) on the test set, respectively. Using Cochran’s Q test, there was no significant differences in the diagnostic level among three STIC-assisted doctors. When comparing the diagnostic level between three STIC-assisted doctors and doctors’ consensus diagnosis, there were significant differences in sensitivity for ICC (*p* value = 0.038). **D** The case study of three test samples pathologically diagnosed with ICC. For case 1, the enhancement pattern of CECT was typical, where ICC tumor showed homogeneously low attenuation on NC phase, faint peripheral enhancement on ART phase and gradual centripetal enhancement on PV phase. The diagnosis of doctors’ consensus was ICC. The output of the STIC model was {HCC: 0.067, ICC: 0.646, metastasis: 0.287}. All three STIC-assisted doctors independently diagnosed it as ICC. For case 2, the enhancement pattern of CECT was similar with the typical pattern of HCC tumor, exhibiting low attenuation on NC phase, the early peak of enhancement on ART phase, and followed by a continuous decrease in PV phase. The doctors’ consensus misdiagnosed it as HCC. The output of the STIC model was {HCC: 0.881, ICC: 0.067, metastasis: 0.052}, which also diagnosed it as HCC incorrectly. All three STIC-assisted doctors misdiagnosed it as HCC. For case 3, there was peripheral enhancement on ART phase, but it was not obvious to the human eyes. The doctors’ consensus misdiagnosed it as metastasis. The output of the STIC model was {HCC: 0.114, ICC: 0.587, metastasis: 0.299}, which diagnosed it as ICC correctly. All three STIC-assisted doctors diagnosed it as ICC correctly. **E** The case study of three test samples pathologically diagnosed with metastasis. For case 1, the doctors’ consensus misdiagnosed it as ICC. The output of the STIC model was {HCC: 0.031, ICC: 0.343, metastasis: 0.626}. Two STIC-assisted doctors independently diagnosed it as metastasis correctly. One STIC-assisted doctor misdiagnosed it as metastasis. For case 2, the doctors’ consensus misdiagnosed it as ICC. The output of the STIC model was {HCC: 0.306, ICC: 0.240, metastasis: 0.454}. All three STIC-assisted doctors independently diagnosed it as metastasis correctly. For case 3, the doctors’ consensus misdiagnosed it as ICC. The output of the STIC model was {HCC: 0.173, ICC: 0.176, metastasis: 0.651}. All three STIC-assisted doctors independently diagnosed it as metastasis correctly. **F** The ROC curve analysis of the STIC model for HCC, ICC, metastasis diagnosis on the external test set for additional verification. The AUC for diagnosis of HCC, ICC and metastasis on the external test set was 0.986, 0.881 and 0.920, respectively. **G** Comparison of the performance of the STIC model on the test set from center 1 and on the external test set from center 2 for differentiating malignant hepatic tumors. Using McNemar’s Chi-squared test, the STIC model’s performance has no significant difference on the center 1 and center 2 for the accuracy, sensitivity and specificity of each type of malignant tumors. Using DeLong test for two ROC curves’ comparison, the STIC mode achieved significant better performance on the external test set from center 2 than on the test set from center 1 for the AUC of HCC diagnosis (*p* value = 0.048) and ICC diagnosis (*p* value = 0.039)

We extended the proposed STIC model to classify three types of malignant hepatic tumors and achieved the total accuracy of 72.6% on the test set. The micro-average and macro-average AUC of the STIC model was 0.868 and 0.852 (Fig. 2A). The AUC for diagnosis of HCC, ICC and metastasis was 0.937, 0.727 and 0.878, respectively (Fig. 2B). We further evaluated the performance of doctors’ consensus diagnosis and model assisted diagnosis on the test set. The total accuracy of the doctors’ consensus was 70.8%, and three STIC-assisted doctors achieved the average accuracy of 79.1%, with an increase of 8.3% than doctors’ consensus (Fig. 2C and Additional file 2: Table S7). There were no significant differences in accuracy, sensitivity and specificity for each type of tumors between the STIC model and doctors’ consensus diagnosis (Fig. 2C and Additional file 2: Table S8), which showed that our proposed STIC model is comparable with human experts’ performance. When comparing the diagnostic level between three STIC-assisted doctors and doctors’ consensus diagnosis, there were significant differences in sensitivity for ICC (*p* value = 0.038) (Fig. 2C and Additional file 2: Table S9). With the assistance of STIC predicted scores, all three doctors achieved higher diagnostic sensitivity for ICC, with an increase of 26.9% on average. In addition to resection of the involved liver, portal lymphadenectomy is recommended for ICC during surgery [10]. The accurate diagnosis for ICC can avoid the risk of skipping portal lymphadenectomy, which is of great clinical value.

## Case study of test samples that doctors initially misdiagnosed

We performed the case study of test samples that doctors initially misdiagnosed to illustrate the process of STIC-assisted diagnosis. We list three cases pathologically diagnosed with ICC (Fig. 2D) and three cases pathologically diagnosed with metastasis (Fig. 2E) on the test set as examples. The enhancement pattern of ICC case 1 was typical for ICC samples, but ICC case 2, 3 represented the ICC samples that have atypical radiological features and were easily misdiagnosed clinically. The scores outputted by the STIC model for ICC case 3 effectively assisted doctors to make an accurate diagnosis, which can guide them specifying the surgical protocol. Clinically, it is important but challenging to differ ICC from metastasis. Metastases 1, 2 and 3 were all misdiagnosed as ICC by doctors’ consensus. With the assistance of our STIC model, doctors were more likely to diagnose them as metastasis correctly. These results show that the cooperation paradigm that combines the experience and knowledge of doctors with our established AI assistance system can provide more accurate differential diagnosis of malignant hepatic tumors.

## Generalization performance of the STIC model on the external test set

On the external test set from center 2, our STIC model achieved an accuracy of 82.9%, the micro-average AUC of 0.944 and the macro-average AUC of 0.931 (Fig. 2F and Additional file 2: Table S10). The accuracy, sensitivity and specificity for each type of malignant tumors have no significant difference on the test set from center 1 and on the external test set from center 2 (Fig. 2G). Using AUC as the evaluation index, our STIC model even achieved significant better performance for HCC and ICC diagnosis on the external test set (Fig. 1G and Additional file 2: Table S10), which may be related to the lower missing rate of clinical data on the external test set (Additional file 2: Table S1). The completeness of preoperative clinical data is expected to further improve the accuracy of our model. The diagnostic performance on the external test set from center 2 verifies the generalization ability of the STIC model. Considering the flexibility of our model’s architecture, the prediction of some prognostic indicators such as MVI for hepatic tumors and differentiation of metastases among distinct primary cancers will be incorporated in our future work.

In conclusion, our proposed deep learning model can differentiate HCC, ICC and metastasis through using deep CNN and gated RNN to integrate multimodal input of multi-phase CECT images and clinical features, with promising performance comparable with experienced doctors and good generalization ability on different centers. Doctors assisted with our model can improve diagnostic performance, especially for the diagnosis of ICC, showing the great potential of AI assistance system in precise diagnosis and treatment of liver cancer.

## Supplementary Information

**Additional file 1.** Supplementary Materials and Methods.

**Additional file 2.** Supplementary Tables.

## Data Availability

The datasets used and/or analyzed during the current study are available from the corresponding author on reasonable request.

## Abbreviations

CECT: Contrast-enhanced computed tomography
HCC: Hepatocellular carcinoma
ICC: Intrahepatic cholangiocarcinoma
CNN: Convolutional neural network
RNN: Recurrent neural network
AI: Artificial intelligence
MVI: Microvascular invasion
