# Supplementary material for: Deep learning for differential diagnosis of malignant hepatic tumors based on multi-phase contrast-enhanced CT and clinical data
Source: J Hematol Oncol. 2021 Sep 26;14:154. doi: 10.1186/s13045-021-01167-2 (PMC8474892; doi:10.1186/s13045-021-01167-2)
Supplement: Supplementary file 1 — Additional file 1. Supplementary Materials and Methods. [file 13045_2021_1167_MOESM1_ESM.docx]

**Supplementary Materials and Methods**

## Dataset

In the preliminary study for differentiating benign and malignant hepatic tumors, 152 pathologically confirmed benign hepatic tumors (hemangioma: 62; FNH: 11; cyst: 75; hepatic hamartoma: 4) and 159 malignant hepatic tumors (HCC: 60; ICC: 35; metastasis: 64) were collected in Xinhua Hospital Affiliated to Shanghai Jiao Tong University School of Medicine. After the preliminary study, we focused on the differential diagnosis for malignant hepatic tumors and collected 723 malignant samples from two centers in total. 612 patients were collected in Xinhua Hospital Affiliated to Shanghai Jiao Tong University School of Medicine (Xinhua cohort) and 111 patients were collected in Affiliated Hangzhou First People’s Hospital, Zhejiang University School of Medicine (Hangzhou cohort). For the two cohorts, inclusion criteria are as following: (1) Pathologically confirmed with one of the following malignant hepatic tumors: HCC, ICC and metastasis; (2) Preoperative multi-phase contrast enhanced CT is available. Exclusion criteria are as following: (1) Age <= 18 years old; (2) Prior liver resection or transplantation; (3) Interval between the pathologic examination and the preoperative CT > 100 days; (4) Poor image quality. The training set consisted of 499 patients who were diagnosed between August 2012 and September 2019 in Xinhua cohort, and the test set consisted of 113 patients who were diagnosed between October 2019 and September 2020 in Xinhua cohort. The external test set consisted of 111 patients in Hangzhou cohort. The patients had signed broad informed consent and this study was approved by the Ethics Committee of Xinhua Hospital Affiliated to Shanghai Jiao Tong University School of Medicine (Approval No. XHEC-D-2021-061).

## CT acquisition protocol

CT scans were performed with a 64-channel MDCT (Somatom Definition, Siemens, Forchheim, Germany). Each patient fasted for more than four hours before undergoing CT scanning. The CT parameters were as follows: detector collimation (1 mm), pitch (0.9), gantry rotation (0.5), tube voltage (120 kV), tube current (240 mA), matrix (512 × 512), slice thickness (5 mm), and reconstruction interval (1 mm). The arterial phase (ART phase), portal venous phase (PV phase), and delay phase (DL phase) scanning were obtained using a fixed 25 sec, 65 sec, and 180 sec equilibrium following intravenous injection of 100 mL of ionic contrast material at a rate of 3 mL/sec using an automatic injector and scan ranging from diaphragm to iliac crest.

## CT images preprocessing

Multi-phase CECT images were exported from Picture Archiving and Communication System (PACS) and stored as Data Interchange Standard for Biomedical Imaging (DICOM) files. Due to the high missing rate of DL phase CT, we excluded this phase in the following image preprocess. 2D ﻿axial slices with the largest tumor area along the z-axis from 3D CT images were selected as the input of diagnostic model. The CT value of original DICOM format images ranged from -1024 Hounsfield unit (HU) to 3071 HU. To focus on the liver part in abdominal CT scans and reduce the noise coming from irrelevant organs, we set Window Width (WW) as 400 and Window Level (WL) as 40. Considering some position deviations among different phase CT images, we chose the non-contrast-enhanced phase (NC phase) as a reference and registered the other phase CT images using the affine registration algorithm. HU value conversion and multi-phase CT registration were implemented with Python 3.6 packages Pydicom 2.0.0 and Airlab respectively. All images were resized to 224 × 224 pixels by interlinear interpolation using OpenCV-python 3.4.0 to fit the format size of our model input.

## Clinical features

Preoperative clinical features of corresponding patients were retrieved from Hospital Information System (HIS), including age, gender, platelet (PLT), total bilirubin (TBIL), alpha fetoprotein (AFP), carbohydrate antigen 19-9 (CA19-9), carcinoembryonic antigen (CEA), carbohydrate antigen 125 (CA125) and hepatitis B surface antigen (HBsAg). Age was divided into five intervals and then encoded into dummy variables with four binary elements. Gender was represented by a binary variable. The rest were biochemical blood indicators of patients before surgery, mapped to categorical variables according to whether normal or missing and then encoded into dummy variables with binary elements.

## Model architecture

We proposed a deep learning model with the modular design of *SpatialExtractor-TemporalEncoder-Integration-Classifier* (STIC), which takes the preprocessed multi-phase CECT images and corresponding encoded clinical features as input, and finally output the score for each type of malignant hepatic tumors. *SpatialExtractor* module is a deep convolutional neural network (CNN) that uses convolutional layers of VGG16 pretrained on ImageNet to extract detailed spatial features of CECT images and applied a trainable convolutional layer with kernel size of 7$\times$7 to output a feature vector of size 128. *TemporalEncoder* module uses a gated recurrent neural network (RNN) to mine the changing pattern among different CECT phases, which is essential for differential diagnosis of hepatic tumors. RNN is appropriate for longitudinal analysis, and gate recurrent unit (GRU) is a straightforward type of recurrent unites without degrading performance. In this module, RNN with one GRU layer is constructed on the outputs of *SpatialExtractor*. The vector size of each GRU cell is 32, and the output of the *TemporalEncoder* module is a feature vector of size 32. In the *Integration* module, the *TemporalEncoder* output is concatenated with the vector of encoded dummy clinical variables, which integrated a fused feature vector of size 52. Finally, in the *Classifier* module, the *Integration* output was passed through the softmax activation function to implement the malignant hepatic tumors classification.

For comparison, we also built two benchmark models, Naïve RGB model and Naïve joint model. Naïve RGB model puts three phases (NC phase, ART phase, and PV phase) CECT into red, green, and blue color channel, respectively, and use convolutional layers of VGG16 pretrained on ImageNet to extract features. Then these extracted features are connected with three fully connected layers (vector size of 512, 128, and 32, respectively). Finally, the classification task was realized with the softmax activation function. Naïve joint model uses the same strategy as Naïve RGB model to handle multi-phase images. In addition, it also incorporates clinical features.

## Model training

During model training, data augmentation dynamically expanded the training data. Each time, the image was randomly rotated within 10 degrees, the upper and lower positions were translated within a 10% range, and the left and right positions were also within 10%. The whole image was also scaled randomly within 10%. Multi-phase CECT of the same patient used the same augmentation mode to ensure consistency of tumor location in images. The augmentation strategy was just for images, and the clinical features remained unchanged.

The STIC model and two benchmark models were all trained with cross-entropy loss function for 50 epochs. The mini-batch size was set to 32, with the consideration of both the speed of training and the computer memory requirement. To avoid overfitting, we performed early stopping based on validation set performance. The training process was interrupted when the loss score of the validation set did not decrease for 20 epochs. The initial learning rate for Adam optimizer was set to 0.001. If no decrease in validation loss score for ten epochs, then the learning rate was reduced by a factor of 0.8 to prevent the loss function bouncing around the minimum.

All models were implemented using Keras (https://keras.io/) package on the Tensorflow (https://www.tensorflow.org) framework (Python 3.6, Keras 2.2.4, Tensorflow 1.12.0). The training and test process were carried out on the DGX-2 node of the Pi 2.0 HPC of Shanghai Jiao Tong University, and the NVIDIA Tesla V100 with 32GB of memory for each GPU was used to accelerate training.

## Model assessment

Our trained models were finalized with weights frozen and then assessed for their performance on the test set and the external test set. We used accuracy, sensitivity, specificity, positive predictive value (PPV), negative predictive value (NPV), F1-score, and area under the receiver operating characteristic curve (AUC) to comprehensively evaluate the diagnostic performance of models. The micro-average AUC and macro-average AUC were calculated for assessing the performance of the multinomial classification model. The 95% confidence intervals (CIs) of accuracy, sensitivity, specificity, PPV, and NPV were calculated using Clopper-Pearson method. The 95% CIs of AUC were calculated with DeLong method.

## Expert evaluation

Two expert radiologists with more than five years of experience were given multi-phase CECT and clinical features of test samples, which were consistent with the model input. They were blind to pathological results and produced a consensus diagnosis. We compared their diagnosis with the results of our STIC model.

To evaluate the performance of model assisted diagnosis, we also conducted a doctor-STIC collaboration study. Three expert radiologists with more than five years of experience were shown multi-phase CECT, clinical features, and predicted scores by the STIC model of samples on the test set, blinded to pathological results. They then independently made final diagnosis on the assistant of the STIC model.

## ﻿Statistical analysis

Comparison of clinical and radiological characteristics among the training set, test set, and external test set was conducted by Pearson’s Chi-squared test for categorical variables, and by ANOVA for continuous variables. McNemar’s Chi-squared test with continuity correction is used to calculate the p-value of the accuracy, sensitivity, and specificity difference between the two models. Cochran’s Q test is used to calculate the p-value of the accuracy, sensitivity and specificity difference among more than two models. All statistical analyses were conducted with R software (version 3.6.1 https://www.r-project.org/)
