# Supplementary material for: Deep learning for differential diagnosis of malignant hepatic tumors based on multi-phase contrast-enhanced CT and clinical data
Source: J Hematol Oncol. 2021 Sep 26;14:154. doi: 10.1186/s13045-021-01167-2 (PMC8474892; doi:10.1186/s13045-021-01167-2)
Supplement: Supplementary file 2 — Additional file 2. Supplementary Tables. [file 13045_2021_1167_MOESM2_ESM.docx]

**Supplementary Tables**

Table S1. Comparison of clinical and radiological characteristics among the training set, test set, and external test set

| Clinical and radiological characteristics | | Training set  n=499 | Test set  n=113 | External test set  n=111 | p-value ^a^ |
| --- | --- | --- | --- | --- | --- |
| Age (year) | x<40 | 36(7.2%) | 2(1.8%) | 1(0.9%) | 0.036 |
|  | 40≤x<50 | 76(15.2%) | 12(10.6%) | 16(14.4%) |  |
|  | 50≤x<60 | 130(26.1%) | 28(24.8%) | 35(31.6%) |  |
|  | 60≤x<70 | 167(33.5%) | 46(40.7%) | 33(29.7%) |  |
|  | x≥70 | 90(18.0%) | 25(22.1%) | 26(23.4%) |  |
| Gender | Male | 336(67.3%) | 70(61.9%) | 80(72.1%) | 0.271 |
|  | Female | 163(32.7%) | 43(38.1%) | 31(27.9%) |  |
| PLT (10^9g/L) | x>100 | 418(83.8%) | 96(85.0%) | 63(56.8%) | <0.001 |
|  | x≤100 | 59(11.8%) | 11(9.7%) | 48(43.2%) |  |
|  | N/A | 22(4.4%) | 6(5.3%) | 0(0.0%) |  |
| TBIL (umol/L) | x<17.1 | 325(65.1%) | 52(46.0%) | 71(64.0%) | <0.001 |
|  | x≥17.1 | 151(30.3%) | 30(26.6%) | 40(36.0%) |  |
|  | N/A | 23(4.6%) | 31(27.4%) | 0(0.0%) |  |
| AFP (umol/L) | x<7 | 313(62.7%) | 75(66.4%) | 61(55.0%) | <0.001 |
|  | 7≤x<400 | 52(10.4%) | 12(10.6%) | 21(18.9%) |  |
|  | x≥400 | 91(18.3%) | 11(9.7%) | 29(26.1%) |  |
|  | N/A | 43(8.6%) | 15(13.3%) | 0(0.0%) |  |
| CA19-9 (U/ml) | x<39 | 268(53.7%) | 58(51.3%) | 68(61.3%) | <0.001 |
|  | x≥39 | 188(37.7%) | 37(32.8%) | 43(38.7%) |  |
|  | N/A | 43(8.6%) | 18(15.9%) | 0(0.0%) |  |
| CEA (ng/ml) | x<10 | 321(64.3%) | 69(61.1%) | 86(77.5%) | <0.001 |
|  | x≥10 | 136(27.3%) | 26(23.0%) | 25(22.5%) |  |
|  | N/A | 42(8.4%) | 18(15.9%) | 0(0.0%) |  |
| CA125 (U/ml) | x<35 | 326(65.3%) | 63(55.8%) | 60(54.1%) | <0.001 |
|  | x≥35 | 116(23.3%) | 25(22.1%) | 14(12.6%) |  |
|  | N/A | 57(11.4%) | 25(22.1%) | 37(33.3%) |  |
| HBsAg | + | 145(29.1%) | 28(24.8%) | 47(42.3%) | <0.001 |
|  | - | 285(57.1%) | 68(60.1%) | 64(57.7%) |  |
|  | N/A | 69(13.8%) | 25(22.1%) | 0(0.0%) |  |
| Tumor type | HCC | 179(35.9%) | 37(32.7%) | 50(45.1%) | 0.030 |
|  | ICC | 115(23.0%) | 21(18.6%) | 30(27.0%) |  |
|  | Metastasis | 205(41.1%) | 55(48.7%) | 31(27.9%) |  |
| Tumor number | 1 | 409(82.0%) | 90(79.7%) | 90(81.1%) | 0.976 |
|  | 2-3 | 50(10.0%) | 12(10.6%) | 12(10.8%) |  |
|  | >3 | 40(8.0%) | 11(9.7%) | 9(8.1%) |  |
| Maximum diameter (mm) |  | 50.6±31.8 | 40.8±27.2 | 44.6±26.7 | 0.004 |
| Necrosis | Yes | 56(11.2%) | 20(17.7%) | 12(10.8%) | 0.146 |
|  | No | 443(88.8%) | 93(82.3%) | 99(89.2%) |  |

Abbreviations: PLT, platelet; TBIL, total bilirubin; AFP, alpha fetoprotein; CA19-9, carbohydrate antigen 19-9; CEA, carcinoembryonic antigen; CA125, carbohydrate antigen 125; HBsAg, hepatitis B surface antigen; HCC, hepatocellular carcinoma; ICC, intrahepatic cholangiocarcinoma

^a^ The p-value is calculated by Pearson’s Chi-squared test for categorical variables, and by ANOVA for continuous variables.

Table S2. Clinical features dummy encoding

| Clinical features | | Categorical variables | Encoded dummy variables |
| --- | --- | --- | --- |
| Age (year) | x<40 | Age1 | [0,0,0,0] |
|  | 40≤x<50 | Age2 | [1,0,0,0] |
|  | 50≤x<60 | Age3 | [0,1,0,0] |
|  | 60≤x<70 | Age4 | [0,0,1,0] |
|  | x≥70 | Age5 | [0,0,0,1] |
| Gender | Male | Male | [1] |
|  | Female | Female | [0] |
| PLT (10^9g/L) | x>100 | Normal | [1,0] |
|  | x≤100 | Abnormal | [0,1] |
|  | N/A | Absence | [0,0] |
| TBIL (umol/L) | x<17.1 | Normal | [1,0] |
|  | x≥17.1 | Abnormal | [0,1] |
|  | N/A | Absence | [0,0] |
| AFP (umol/L) | x<7 | Normal | [1,0,0] |
|  | 7≤x<400 | Abnormal1 | [0,1,0] |
|  | x≥400 | Abnormal2 | [0,0,1] |
|  | N/A | Absence | [0,0,0] |
| CA19-9 (U/ml) | x<39 | Normal | [1,0] |
|  | x≥39 | Abnormal | [0,1] |
|  | N/A | Absence | [0,0] |
| CEA (ng/ml) | x<10 | Normal | [1,0] |
|  | x≥10 | Abnormal | [0,1] |
|  | N/A | Absence | [0,0] |
| CA125 (U/ml) | x<35 | Normal | [1,0] |
|  | x≥35 | Abnormal | [0,1] |
|  | N/A | Absence | [0,0] |
| HBsAg | + | Positive | [1,0] |
|  | - | Negative | [0,1] |
|  | N/A | Absence | [0,0] |

Abbreviations: PLT, platelet; TBIL, total bilirubin; AFP, alpha fetoprotein; CA19-9, carbohydrate antigen 19-9; CEA, carcinoembryonic antigen; CA125, carbohydrate antigen 125; HBsAg, hepatitis B surface antigen

Table S3. The type and number of benign and malignant hepatic tumors in the preliminary study

| Tumor type | | Sample size |
| --- | --- | --- |
| Benign hepatic tumors | Hepatic hemangioma | 62 |
|  | FNH | 11 |
|  | Hepatic cyst | 75 |
|  | Hepatic hamartoma | 4 |
|  | Total | 152 |
| Malignant hepatic tumors | HCC | 60 |
|  | ICC | 35 |
|  | Metastasis | 64 |
|  | Total | 159 |

Abbreviations: FNH, focal nodular hyperplasia; HCC, hepatocellular carcinoma; ICC, intrahepatic cholangiocarcinoma

Table S4. Performance of the STIC model for classifying benign and malignant hepatic tumors in the preliminary study

| Metric | 5-fold cross validation | | | | | Mean |
| --- | --- | --- | --- | --- | --- | --- |
|  | Fold 1 | Fold 2 | Fold 3 | Fold 4 | Fold 5 |  |
| Accuracy | 0.968 | 0.968 | 0.919 | 0.871 | 0.934 | 0.932±0.040 |
| Sensitivity | 0.969 | 1.000 | 0.875 | 0.781 | 1.000 | 0.925±0.095 |
| Specificity | 0.968 | 0.935 | 0.967 | 0.967 | 0.867 | 0.941±0.044 |
| F1-score | 0.969 | 0.970 | 0.918 | 0.862 | 0.939 | 0.932±0.045 |
| AUC | 0.998 | 1.000 | 0.974 | 0.984 | 1.000 | 0.987±0.010 |

Abbreviations: AUC, area under the Receiver Operating Characteristic curve

Note: sensitivity and specificity are defined by viewing malignant tumors as positive and benign tumors as negative; ﻿the mean AUC of 5-fold cross validation is calculated by the area under the mean Receiver Operating Characteristic curve

Table S5. Performance of the STIC model and two benchmark models for binary classification of primary malignant hepatic tumors on the test set

| Metric | Model types | | |
| --- | --- | --- | --- |
|  | STIC model | Naïve RGB model | Naïve joint model |
| Accuracy | 0.862 (0.746-0.939) | 0.603 (0.466-0.730) | 0.690 (0.555-0.805) |
| Sensitivity | 0.892 (0.746-0.970) | 0.622 (0.448-0.775) | 0.703 (0.530-0.841) |
| Specificity | 0.810 (0.581-0.946) | 0.571 (0.340-0.782) | 0.667 (0.430-0.854) |
| F1-score | 0.892 | 0.667 | 0.743 |
| AUC | 0.893 (0.803-0.982) | 0.709 (0.573-0.845) | 0.766 (0.644-0.888) |

Abbreviations: PPV, positive predictive value; NPV, negative predictive value; AUC, area under the Receiver Operating Characteristic curve

Note: ﻿data in parentheses are 95% confidence intervals

Table S6. Performance of models using different extractor’s backbone for differentiating primary malignant hepatic tumors

| Model performance | Extractor’s backbone | | | | | p-value ^a^ |
| --- | --- | --- | --- | --- | --- | --- |
|  | VGG-Net | ResNet | Xception | Inception | Inception-ResNet |  |
| STIC model |  |  |  |  |  |  |
| Accuracy | 0.862(0.746-0.939) | 0.793(0.666-0.888) | 0.845(0.726-0.927) | 0.793(0.666-0.888) | 0.845(0.726-0.927) | 0.390 |
| Sensitivity | 0.892(0.746-0.970) | 0.838(0.680-0.938) | 0.865(0.712-0.955) | 0.892(0.746-0.970) | 0.892(0.746-0.970) | 0.794 |
| Specificity | 0.810(0.581-0.946) | 0.714(0.478-0.887) | 0.810(0.581-0.946) | 0.619(0.384-0.819) | 0.762(0.528-0.918) | 0.113 |
| Naïve RGB model |  |  |  |  |  |  |
| Accuracy | 0.603(0.466-0.730) | 0.414(0.286-0.551) | 0.603(0.466-0.730) | 0.534(0.399-0.667) | 0.621(0.484-0.745) | 0.136 |
| Sensitivity | 0.622(0.448-0.775) | 0.189(0.080-0.352) | 0.730(0.559-0.862) | 0.405(0.248-0.579) | 0.703(0.530-0.841) | <0.001 |
| Specificity | 0.571(0.340-0.782) | 0.810(0.581-0.946) | 0.381(0.181-0.616) | 0.762(0.528-0.918) | 0.476(0.257-0.702) | 0.012 |
| Naïve joint model |  |  |  |  |  |  |
| Accuracy | 0.690(0.555-0.805) | 0.621(0.484-0.745) | 0.707(0.573-0.819) | 0.690(0.555-0.805) | 0.638(0.501-0.760) | 0.808 |
| Sensitivity | 0.703(0.530-0.841) | 0.432(0.271-0.605) | 0.946(0.818-0.993) | 0.595(0.421-0.752) | 0.432(0.271-0.605) | <0.001 |
| Specificity | 0.667(0.430-0.854) | 0.952(0.762-0.999) | 0.286(0.113-0.522) | 0.857(0.637-0.970) | 1.000(0.839-1.000) | <0.001 |

^a^ The p-value is calculated by Cochran’s Q test.

Table S7. Comparison of malignant liver tumors diagnostic performance of the STIC model, doctors’ consensus and three doctors assisted by the STIC model on the test set

|  | Diagnosis methods | | | | |
| --- | --- | --- | --- | --- | --- |
| Metric | STIC model | Doctors’ consensus | STIC-assisted | | |
|  |  |  | Doctor 1 | Doctor 2 | Doctor 3 |
| Accuracy | 0.726(0.634-0.805) | 0.708(0.615-0.790) | 0.770(0.681-0.844) | 0.788(0.701-0.859) | 0.814(0.730-0.881) |
| HCC |  |  |  |  |  |
| Sensitivity | 0.865(0.712-0.955) | 0.784(0.618-0.902) | 0.811(0.648-0.920) | 0.784(0.618-0.902) | 0.919(0.781-0.983) |
| Specificity | 0.868(0.771-0.935) | 0.947(0.871-0.985) | 0.908(0.819-0.962) | 0.934(0.853-0.978) | 0.921(0.836-0.970) |
| PPV | 0.762(0.605-0.879) | 0.879(0.718-0.966) | 0.811(0.648-0.920) | 0.853(0.689-0.950) | 0.850(0.702-0.943) |
| NPV | 0.930(0.843-0.977) | 0.900(0.812-0.956) | 0.908(0.819-0.962) | 0.899(0.810-0.955) | 0.959(0.885-0.991) |
| F1-score | 0.810 | 0.829 | 0.811 | 0.817 | 0.883 |
| AUC | 0.937(0.892-0.982) | N/A | N/A | N/A | N/A |
| ICC |  |  |  |  |  |
| Sensitivity | 0.429(0.218-0.660) | 0.429(0.218-0.660) | 0.762(0.528-0.918) | 0.619(0.384-0.819) | 0.714(0.478-0.887) |
| Specificity | 0.859(0.770-0.923) | 0.815(0.721-0.889) | 0.870(0.783-0.931) | 0.880(0.796-0.939) | 0.902(0.822-0.954) |
| PPV | 0.409(0.207-0.636) | 0.346(0.172-0.557) | 0.571(0.372-0.755) | 0.542(0.328-0.744) | 0.625(0.406-0.812) |
| NPV | 0.868(0.781-0.930) | 0.862(0.771-0.927) | 0.941(0.868-0.981) | 0.910(0.851-0.960) | 0.933(0.859-0.975) |
| F1-score | 0.419 | 0.383 | 0.653 | 0.578 | 0.667 |
| AUC | 0.727(0.599-0.855) | N/A | N/A | N/A | N/A |
| Metastasis |  |  |  |  |  |
| Sensitivity | 0.745(0.610-0.853) | 0.764(0.630-0.868) | 0.745(0.610-0.853) | 0.855(0.733-0.935) | 0.782(0.650-0.882) |
| Specificity | 0.862(0.746-0.939) | 0.793(0.666-0.888) | 0.879(0.767-0.950) | 0.862(0.746-0.939) | 0.897(0.788-0.961) |
| PPV | 0.837(0.703-0.927) | 0.778(0.644-0.880) | 0.854(0.722-0.939) | 0.855(0.733-0.935) | 0.878(0.752-0.954) |
| NPV | 0.781(0.660-0.875) | 0.780(0.652-0.877) | 0.785(0.665-0.877) | 0.862(0.746-0.939) | 0.813(0.695-0.899) |
| F1-score | 0.788 | 0.771 | 0.796 | 0.855 | 0.827 |
| AUC | 0.878(0.816-0.940) | N/A | N/A | N/A | N/A |

Abbreviations: PPV, positive predictive value; NPV, negative predictive value; AUC, area under the Receiver Operating Characteristic curve Note: ﻿data in parentheses are 95% confidence intervals

Table S8. Comparison of the STIC model and doctors’ consensus diagnosis in differentiating HCC, ICC and metastasis on the test set

| STIC vs. Doctors’ consensus | Difference (95% CI) | p-value ^a^ |
| --- | --- | --- |
| Accuracy | 0.018 (-0.089,0.125) | 0.860 |
| HCC |  |  |
| Sensitivity | 0.081(-0.120,0.282) | 0.546 |
| Specificity | -0.079(-0.163,0.005) | 0.077 |
| ICC |  |  |
| Sensitivity | 0(-0.234,0.234) | 0.617 |
| Specificity | 0.044(-0.067,0.154) | 0.522 |
| Metastasis |  |  |
| Sensitivity | -0.018(-0.183,0.147) | 1 |
| Specificity | 0.069(-0.054,0.192) | 0.343 |

^a^ The p-value is calculated by McNemar’s Chi-squared test with continuity correlation

Table S9. Diagnostic level comparison of three STIC-assisted doctors and doctors’ consensus on the test set

|  | Average of three STIC-assisted doctors | p-value among three STIC-assisted doctors ^a^ | p-value among three doctors and  doctors’ consensus diagnosis ^a^ |
| --- | --- | --- | --- |
| Accuracy | 0.791 | 0.581 | 0.131 |
| HCC |  |  |  |
| Sensitivity | 0.838 | 0.123 | 0.236 |
| Specificity | 0.921 | 0.741 | 0.644 |
| ICC |  |  |  |
| Sensitivity | 0.698 | 0.368 | 0.038 |
| Specificity | 0.884 | 0.692 | 0.214 |
| Metastasis |  |  |  |
| Sensitivity | 0.794 | 0.211 | 0.357 |
| Specificity | 0.879 | 0.717 | 0.224 |

Abbreviations: HCC, hepatocellular carcinoma; ICC, intrahepatic cholangiocarcinoma;

^a^ The p-value is calculated by Cochran’s Q test

Table S10. Performance of the STIC model for multinomial classification of malignant hepatic tumors on the test set and external test set

|  | Test set from the center 1 | External test set from the center 2 | p-value between two centers ^a^ | Total of two centers |
| --- | --- | --- | --- | --- |
| Accuracy | 0.726(0.634-0.805) | 0.829(0.746-0.894) | 0.090 | 0.777(0.717-0.830) |
| HCC |  |  |  |  |
| Sensitivity | 0.865 (0.712-0.955) | 0.980(0.894-0.999) | 0.095 | 0.931(0.856-0.974) |
| Specificity | 0.868 (0.771-0.935) | 0.918(0.819-0.973) | 0.516 | 0.891(0.826-0.937) |
| AUC | 0.937 (0.892-0.982) | 0.986(0.968-1.000) | 0.048 | 0.968(0.948-0.988) |
| ICC |  |  |  |  |
| Sensitivity | 0.429 (0.218-0.660) | 0.667(0.472-0.827) | 0.161 | 0.569(0.422-0.707) |
| Specificity | 0.859 (0.770-0.923) | 0.914(0.830-0.965) | 0.374 | 0.884(0.827-0.928) |
| AUC | 0.727 (0.599-0.855) | 0.881(0.812-0.949) | 0.039 | 0.809(0.742-0.876) |
| Metastasis |  |  |  |  |
| Sensitivity | 0.745 (0.610-0.853) | 0.742(0.554-0.881) | 1.000 | 0.744(0.639-0.832) |
| Specificity | 0.862 (0.746-0.939) | 0.913(0.828-0.964) | 0.508 | 0.891(0.827-0.938) |
| AUC | 0.878 (0.816-0.940) | 0.920(0.869-0.971) | 0.316 | 0.900(0.861-0.939) |

Abbreviations: HCC, hepatocellular carcinoma; ICC, intrahepatic cholangiocarcinoma; AUC, area under the Receiver Operating Characteristic curve

^a^ The p-value is calculated by McNemar’s Chi-squared test with continuity correlation for accuracy, sensitivity and specificity, and by DeLong test for AUC.

**Supplementary Figures**


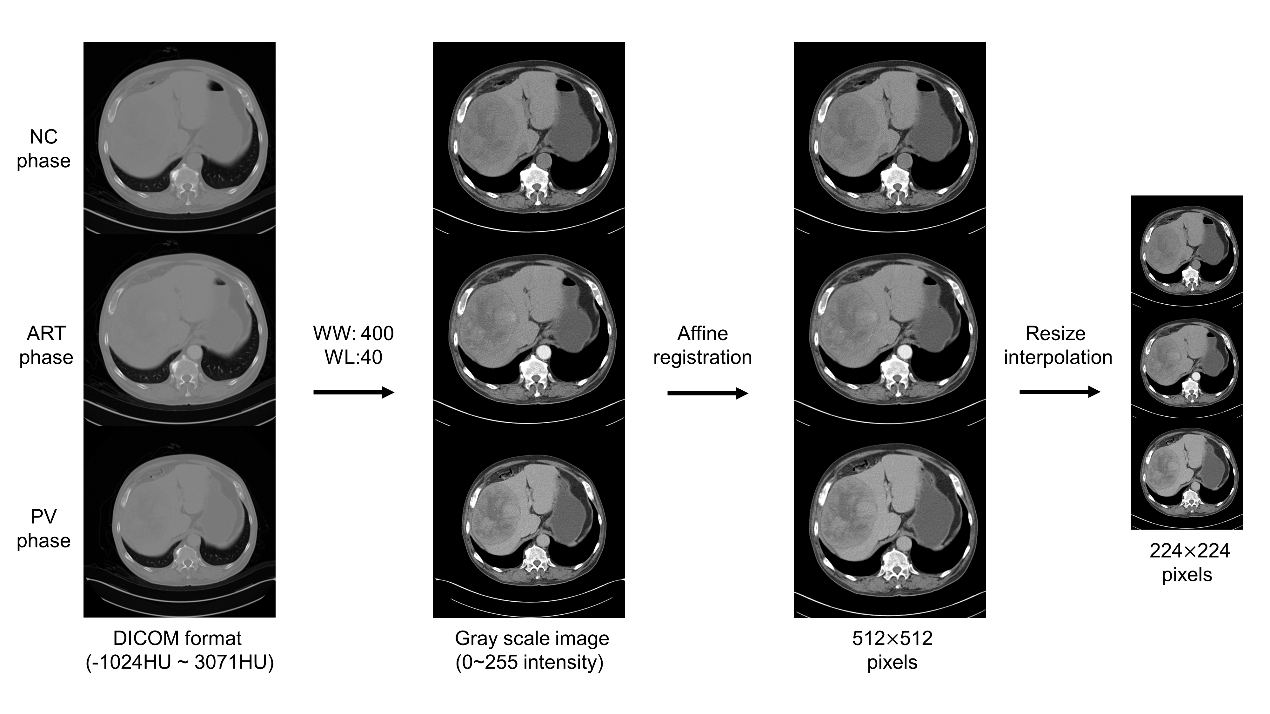


Figure S1. The workflow of CT images preprocessing. The CT value of original DICOM format images ranged from -1024 Hounsfield unit (HU) to 3071 HU. We set Window Width (WW) as 400 and Window Level (WL) as 40 to make HU value conversion. Then we chose the non-contrast-enhanced phase (NC phase) as a reference and registered the other phase CT images using the affine registration algorithm. Lastly, all images were resized to 224 × 224 pixels by interlinear interpolation to fit the format size of our model input.


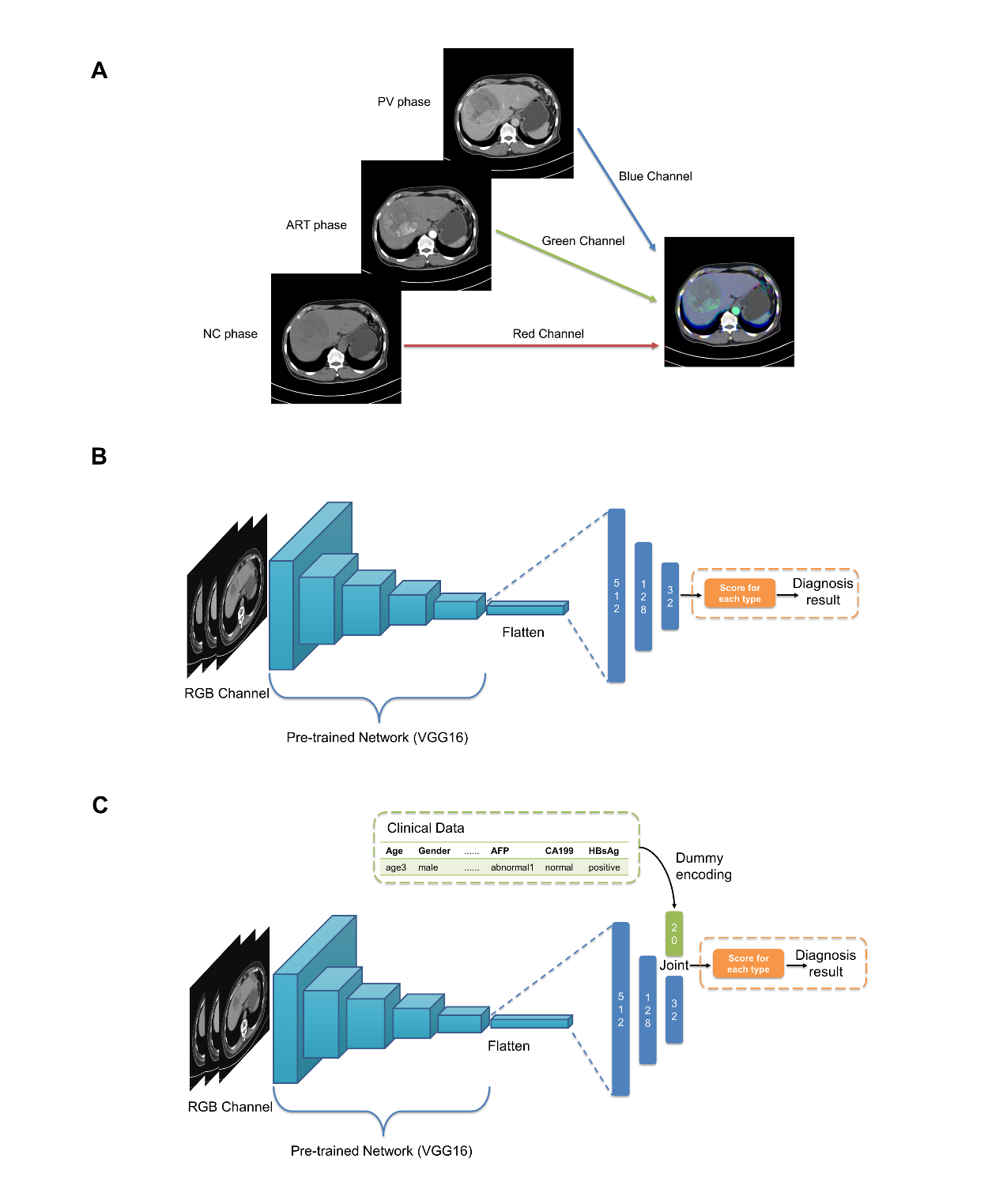


Figure S2. The details of two benchmark models. **A** Putting non-contrast-enhanced phase (NC phase), arterial phase (ART phase) and portal venous phase (PV phase) CECT images into red, green and blue color channel, respectively. **B** The architecture of the Naïve RGB model. It uses convolutional layers of VGG16 pretrained on ImageNet to extract features. Then these extracted features are connected with three fully connected layers. Finally, the classification task is realized with the softmax activation function. **C** The architecture of the Naïve joint model. It uses the same strategy as Naïve RGB model to handle multi-phase images. In addition, it also incorporates clinical data to make a final classification.
